# Surgical resection of recurrent intramedullary subependymoma of the cervical spinal cord

**DOI:** 10.3171/2023.7.FOCVID2394

**Published:** 2023-10-01

**Authors:** Jennifer L. Perez, Maria D. Astudillo Potes, Juliana C. Rotter, Megan C. Everson, Aditya Raghunathan, Michelle J. Clarke

**Affiliations:** Departments of 1 Neurosurgery and; 2 Pathology, and; 3 Alix School of Medicine, Mayo Clinic, Rochester, Minnesota

**Keywords:** spinal subependymoma, intramedullary spinal tumor, rare spinal tumors

## Abstract

Spinal subependymomas (SE) are rare, often indolent benign tumors presenting most frequently as intramedullary tumors in the cervical spine or cervicothoracic junction. When symptomatic, patients often present with years of sensory changes, weakness, paresthesias, or bowel and bladder dysfunction. Preoperatively, SE are difficult to distinguish radiographically from ependymomas or astrocytomas; however, it is important to make the distinction intraoperatively as complete resection can be curative. Here the authors present a rare case of recurrent, symptomatic cervical subependymoma which underwent gross-total resection and discussion of management strategies and outcomes of all SE at their institution.

## Transcript

Here we present the surgical resection of a recurrent intramedullary subependymoma of the cervical spinal cord.

### 0:30 Clinical Presentation and Neurological Exam.

This is the case of a 34-year-old male with a history of morbid obesity and a known cervical subependymoma for which he underwent a previous C2–7 laminectomy, tumor resection, and C2–T4 fusion in 2016. Postoperatively, he has significant left arm paresis, with partial regain of function following rehabilitation, but was subsequently lost to follow-up for several years.

### 1:00

His initial preoperative imaging showed an extensive intramedullary lesion from C2 to 7 with minimal heterogenous T1 contrast enhancement seen in the top left and minimal T2 signal in the top middle.^1^ Within the limitations of instrumentation artifact, postoperative imaging in the bottom panel showed no evidence of abnormal contrast enhancement within the cord or the thecal sac and excellent decompression of the spinal cord.

### 1:26 History of Present Illness Continued.

He returned in 2023 with 1 year of progressive left arm weakness and gait instability. By the time he presented, he had near-complete absence of motor and sensory function in the left arm.

### 1:40 Preoperative Imaging.

Repeat cervical MRI with contrast showed expansile intramedullary lesion from C3 to 7 with the largest bulk centered at C5–6. There is also T2 hyperintensity in the edge of the dorsal lateral left hemicord up to the C2 level and down to the T1 level compatible with myelomalacia.

### 2:03

CT confirmed solid bony fusion and decision was made to remove hardware during the case to improve visualization and enhance the accuracy of tumor assessment on follow-up imaging.

### 2:14 Key Surgical Steps.

Patient was placed in prone position with the head secured in pinions. Previous midline cervical incision was identified and reopened in typical fashion. Neuromonitoring with SSEPs (somatosensory evoked potentials) and MEPs (motor evoked potentials) was utilized during the case.

### 2:31 Tumor Identification and Dissection.

Given the difficulty visualizing the tumor on MRI, ultrasound was used to identify the boundaries of the tumor.

### 2:42

Despite scarring, the midline of the spinal cord was located based on cervical surface topography. Moving through the posterior median septum resulted in minimal spinal cord damage as a clear plane was found between the gracilis fasciculi.

### 2:57

The tumor was identified and separated from the spinal cord by spreading plate forceps.

### 3:15 Tumor Resection.

This demonstrates one plate being used for gentle retraction on the cord and the other applying pressure predominantly to the tumor mass, lessening the trauma to the cord.

### 3:41

Here we show removal of the final pieces of tumor that were adherent to the cord. Ultimately, a gross-total resection was achieved and the areas of adherence gently cauterized.

### 4:05

Ultrasound was used to confirm gross-total resection.

### 4:09 Closure Technique.

A primary watertight dural closure was completed.

### 4:17 Clinical Outcome.

In the immediate postoperative period, the patient had new weakness in his right arm and hand as well as bilateral legs that correlated with intraoperative neuromonitoring changes.

### 4:30

He was admitted to the Neuro ICU for MAP augmentation. His course was complicated by elevated CK (creatinine kinase) secondary to rhabdomyolysis. This was successfully treated without kidney injury. He was transferred to the general care floor on postoperative day 4.

### 4:57

Baseline postoperative cervical MRI showed interval removal of C2–T4 instrumentation and gross-total resection of intramedullary lesion within the cervical cord. Similar T2 hyperintensity and left hemicord atrophy remained. There is an immediate postoperative fluid collection that is expected to resolve over time.

### 5:08

The patient was discharged to inpatient rehab on postoperative day 9. At the time of discharge, his motor exam was improving, and he was regaining function of his right hand.

### 5:19 Final Pathology.

Final pathology showed clinically recurrent ependymal neoplasm with predominantly subependymoma morphology. It had low proliferative activity without significant anaplastic histological features, such as TERT promoter mutation or any chromosomal copy number alterations.^2^

### 5:36 Case Series.

A total of 4 patients were identified in our electronic medical record system with biopsy-confirmed intramedullary spinal subependymoma, and 1 patient with presumed subependymoma. Of the biopsy-confirmed patients, the average age of the patient was 40 years old with equal male to female ratio. Two patients had cervical thoracic lesions and two with isolated thoracic lesions. The patients had an average of 1.4 years of symptom progression prior to presentation, with sensory changes being the most common presenting symptom. Patients 1–4 underwent surgical management that included laminectomies and tumor resection of the respective levels. Given patient 5 was asymptomatic from the lesion, they were observed for a period of several years without progression.

### 6:24

Gross-total resection was achieved in 3 of 4 patients. All patients experienced new postoperative deficits with at least partial recovery. Late tumor recurrence occurred in all cases with an average time to progression of 6.8 years.

### 6:40 Conclusions.

In conclusion, spinal subependymomas are rare tumors that are difficult to visualize and distinguish on imaging.^1–5^ However, intraoperative ultrasound proved to be a useful technique to define the boundaries of the tumor. If feasible, gross-total resection can have a favorable outcome, though late recurrence is known to occur.

## Disclosures

The authors report no conflict of interest concerning the materials or methods used in this study or the findings specified in this publication.

## Author Contributions

Assistant surgeon: Perez, Rotter, Everson. Editing and drafting the video and abstract: Clarke, Perez, Astudillo Potes, Rotter, Everson. Critically revising the work: Clarke, Perez, Everson, Raghunathan. Reviewed submitted version of the work: Clarke, Perez, Astudillo Potes, Everson, Raghunathan. Approved the final version of the work on behalf of all authors: Clarke. Supervision: Clarke, Perez, Raghunathan.

## Supplemental Information

### Patient Informed Consent

Informed consent was waived due to minimal risk to research subjects.
